# Characterization of Type I and Type III Collagen in the Intramuscular Connective Tissue of Wuzhumuqin Sheep

**DOI:** 10.3390/ani13030395

**Published:** 2023-01-24

**Authors:** Xige He, Qiong Wu, Wenjun Xue, Rihan Wu, Yajuan Huang, Lu Chen, Yunfei Han, Jindi Wu, Gerelt Borjigin, Rina Sha

**Affiliations:** 1 College of Food Science and Engineering, Inner Mongolia Agricultural University, Hohhot 010018, China; 2 Ke Er Qin You Yi Front Banner Administration for Market Regulation, Xing’an League 137400, China; 3 College of Biochemistry and Engineering, Hohhot Vocational College, Hohhot 010051, China

**Keywords:** *Wuzhumuqin sheep*, intramuscular connective tissue, collagen, amino acid, cross-linkage

## Abstract

**Simple Summary:**

Collagen is an important component of muscle tissue that maintains muscle structure and also contributes to meat quality. However, the characteristics of intramuscular connective tissue (IMCT) collagen of *Wuzhumuqin sheep* are unknown. The purpose of this study was to analyze the characteristics of IMCT collagen in *Wuzhumuqin sheep* at different growth stages and to determine the correlation between them. The results showed that collagen-related genes were highly expressed at 9 and 12 months of age. The amino acid content, solubility, thermal stability, and degree of cross-linking of collagen were altered by collagen type, muscle type, and age. Multiple patterns of correlation between collagen characteristics are essential for the development of IMCT. These findings will contribute to a better understanding of the relationship between the characteristics of collagen, and also provide a theoretical basis for further studies on the impacts of collagen on meat quality.

**Abstract:**

Intramuscular connective tissue (IMCT) collagen is an important factor in meat quality. This study analyzed the characteristics of type I and III collagen in the IMCT of the semitendinosus (SD) and longissimus dorsi (LD) of *Wuzhumuqin sheep* at different growth stages (6, 9, 12, and 18 months). Utilizing sodium dodecyl sulfate-polyacrylamide gel electrophoresis (SDS-PAGE) and Fourier transform infrared spectroscopy (FTIR), collagen types I and III were successfully isolated and shown to contain an intact triple helix structure. Immunofluorescence revealed that these collagens were located in the endomysium and perimysium. Collagen-related genes were significantly expressed in sheep aged 9 and 12 months. The amino acid content increased with age in type I collagen whereas it decreased in type III collagen. Furthermore, type III collagen contained more hydroxyproline (Hyd) than type I collagen. Differential scanning calorimetry (DSC) revealed that the thermal stability of collagen increased with age, accompanied by a decrease in solubility. Semitendinosus muscle had more collagen cross-linkages than LD muscle due to the high pyridinoline (Pyr) content in the endomysium. Finally, a correlation analysis highlighted the multiple correlations between characteristics in different types of collagen during sheep growth. In summary, the collagen characteristics in the IMCT of sheep were impacted by collagen type, muscle type, and age. Furthermore, the various correlations between these characteristics may play an important role in the development of IMCT.

## 1. Introduction

Connective tissue is a critical structural component of muscle. It maintains the structural integrity of muscle fibers and functions as a kinematic system [1]. The intramuscular connective tissue (IMCT) of skeletal muscle is primarily made up of epimysium, perimysium, and endomysium. As the epimysium is usually thick and tough, resistant to shearing and dissolution, and easily separated from the muscle when eaten, the connective tissues that affect muscle tenderness are mainly the endomysium and perimysium. The endomysium and perimysium form a three-dimensional network that acts as a link, support, and force transmitter in the muscle; it also has a complex relationship with meat tenderness and is the main source of intrinsic muscle stiffness [2].

Collagen, a major component of IMCT, maintains muscle structure and is also important in determining meat quality. In a previous study, young male beef from 15 European breeds was analyzed to determine the relationship between collagen characteristics, fat content, and the quality of raw and cooked meat. Both the collagen content and insoluble collagen content were positively correlated with shear force in raw muscle but not in cooked meat [3]. Similarly, another study found that total collagen in pigs was positively correlated with shear [4]. According to one report, a decrease in muscle shear in bulls with age may be due to a decrease in collagen solubility [5]. In general, the tenderness and acceptability of the meat can be affected by the animal’s age and muscle type. A previous study of lambs slaughtered at 90, 130, and 175 days revealed that shear force increased with age [6]. Different muscles respond differently to aging, which can be partially explained by the various collagen characteristics and protein cross-linking. The solubility of collagen differs among various beef components, being higher in the longest dorsal muscle than in the infraspinatus and semimembranosus muscles [7]. Differential scanning calorimetry (DSC) has been used to analyze the thermal degeneration of connective tissue in various regions of the pork muscle, showing that the thermal degeneration of connective tissue varies [8]. Recent research in pigs suggests that the biceps femoris is stiffer than the longissimus thoracis et lumborum because it contains more total collagen and insoluble collagen [9]. Another study showed that with increasing age, collagen stability cross-linking in muscles and the thermal insolubility of collagen increases, while muscle tenderness decreases [10]. The pattern of pyridine cross-linking in connective tissue in cattle is as follows: semitendinosus (SD) > longest dorsal muscle > ulnar carpal extensor [11].

Collagen is functionally and structurally diverse. Twenty-six different types of collagen have been identified, and are found in a variety of tissues throughout the animal body [12]. The endomysium and fascia contain significant concentrations of type I and type III fibrillar collagen, and play an important role in the structure and function of skeletal muscle. Pork tenderness and the percentage of type III fibrillar collagen are positively correlated [9]. Similar results have been reported for bovine muscle [13]. In addition, as an animal ages, type I and type III collagen not only change in content but also switch between types, which is useful for researching the histological characteristics of collagen as an animal grows. However, few studies have investigated collagen in muscle tissue in sheep, particularly in *Wuzhumuqin sheep*.

*Wuzhumuqin sheep* is a breed of Mongolian sheep that produces high-quality meat. Due to its large body size, high live weight, large meat production, and high meat quality, the majority of mutton consumed by local residents comes from mature sheep. *Wuzhumuqin sheep* graze naturally on the Xilinguole steppe and are accustomed to autonomous activity and completely voluntary foraging. We have previously described the process of skeletal muscle development in *Wuzhumuqin sheep* at various stages of growth and development, at both the cellular and myofiber levels [14,15]. However, to the best of our knowledge, no studies have been conducted on collagen in the muscle tissue of *Wuzhumuqin sheep* at various stages of development. This study concentrated on type I and III collagen in the IMCT in different growth stages of *Wuzhumuqin sheep* and examined the gene expression, collagen distribution, amino acid fraction, denaturation temperature, thermal solubility, degree of cross-linking, and the correlations between these factors. Our findings help explain the properties of collagen in IMCT. This is critical for understanding the relationship between the properties of IMCT and the physicochemical properties of meat and will inform future research on the effect of collagen on meat quality.

## 2. Materials and Methods

### 2.1. Sample Collection

The raw materials for this experiment were obtained from castrated rams in four different growth stages: 6 (6 M, *n* = 15), 9 (9 M, *n* = 15), 12 (12 M, *n* = 15), and 18 (18 M, *n* = 15) months of age. All sheep were naturally grazing in the East Wuzhumuqin region of Inner Mongolia. After slaughter at the local abattoir, the SD and longissimus dorsi (LD) were sampled, frozen in liquid nitrogen, and stored at −80 °C. Samples for indirect immunofluorescence staining were cut into 0.5 cm^3^ pieces, dehydrated in isopentane, quickly frozen in liquid nitrogen, and returned to storage at −80 °C.

### 2.2. Expression Analysis of Genes Related to Collagen Type I and III

Expression analysis of collagen-related genes was performed using a method developed by Livak et al. [16]. Total RNA was extracted from 100 mg muscle tissue using Trizol reagent (Invitrogen, Carlsbad, CA, USA) following the manufacturer’s recommendations. Agarose gel electrophoresis (Thermo Fisher Scientific, Waltham, MA, USA) was used to determine the purity of total RNA, and RNA quality and quantity were analyzed according to their OD_260_/OD_280_ ratios. After isolation, the total RNA was reverse-transcribed by a cDNA reverse transcription kit (Vazyme, Nanjing, China). The expression level of the COL1A1, COL1A2, and COL3A1 genes was examined using ChamQ Universal SYBR qPCR Master Mix (Vazyme, Nanjing, China), with β-actin used as the reference gene (Table S1). Real-time quantitative polymerase chain reaction (qPCR) was performed in a Cycler I Q real-time PCR detection system (Bio-Rad, Hercules, CA, USA), and the relative expression of mRNA was calculated using the 2^−ΔΔCT^ method.

### 2.3. Extraction of Type I and III Collagen

The extraction method was based on Chandrarajan [17] with some modifications. To prepare a washing solution 3.582 g NaHO_4_–12H_2_O was weighed and dissolved, then 1 mL N-maleamide (12.5 μg/mL), 1 mL PMSF (10 mM), and 5 mL EDTA (0.2 M) were added. Distilled water was added to a volume of 1000 mL and the solution was set aside at 4 °C. For the extraction solution, a 1 mg/mL pepsin–0.5 M acetic acid solution was prepared and pepsin was added at a ratio of 1:100. A 2 M guanidine hydrochloride solution was prepared by weighing 19.106 g guanidine hydrochloride, dissolving 0.7882 g Tris-HCI in distilled water, and then adjusting the volume to 100 mL and pH to 7.5.

A 100 g sample of muscle tissue was broken up and homogenized in 10% NaCl-Tris-HCl (pH 7.5) buffer in an ice bath and centrifuged at 10,000× *g* for 15 min; then the precipitate was collected. The homogenate was stirred for 24 h and centrifuged at 5000× *g* for 15 min; then the precipitate was collected and washed by repeated centrifugation with 10 times the volume of washing solution with agitation (5000× *g* for 15 min); and then the precipitate was agitated in 10 times the volume of extraction solution for 48 h at 15 °C. The precipitate was separated after centrifugation at 100,000× *g* for 40 min, the supernatant was collected, NaCl was added to make a concentration of 2 M, and the sample was stirred overnight (12 h). Next, the sample was centrifuged at 10,000× *g* for 40 min and the precipitate was repeatedly dissolved by adding 10 times the amount of 0.5 M acetic acid to the precipitate. After centrifugation at 10,000× *g* for 40 min, NaCl was added to the supernatant to make a 1 M concentration and it was stirred overnight (12 h). The sample was centrifuged again at 10,000× *g* for 1 h and the precipitate (containing type I and III collagen eggs) was repeatedly dissolved in 0.5 M acetic acid, followed by dialysis in 0.45 M NaCl-5 mM Tris-HCl for 12 h and dialysis in 1.2 M NaCl-5 mM Tris-HCl 12 h; then the sample was centrifuged at 12,000× *g* for 1 h. The supernatant that was subsequently obtained was type I collagen, while the precipitate was crude type III collagen, which was subsequently dialyzed in 5 mM acetic acid for 12 h.

The crude type III collagen was further purified. The crude type III collagen was dissolved in a 2 M guanidine hydrochloride solution to a concentration of 0.02–0.1% in a water bath at 45 °C for 30 min and then centrifuged at 3000× *g* for 40 min. The supernatant was diluted in distilled water to a final concentration of 250 μg/mL, dialyzed in distilled water for 2 h, and centrifuged at 100,000× *g* for 1 h to extract the precipitate; then the supernatant was subsequently dialyzed for 16 h. The precipitate was rinsed in distilled water and centrifuged (100,000× *g* for 1 h) to obtain type III collagen.

### 2.4. Sodium Dodecyl Sulfate-Polyacrylamide Gel Electrophoresis (SDS-PAGE) of Type I and III Collagen

SDS-PAGE analysis of type I and III collagen was performed according to the method proposed by Laemmli [18] with the necessary modifications. The solubilized collagen sample was mixed with the loading buffer (1 M Tris-HCl, pH 6.8, 50% propanetriol, β-mercaptoethanol, 1% bromophenol blue, 10% SDS, and 50% glycerin) at a ratio of 4:1 (*v*/*v*). Then 10 μL sample per well was loaded onto polyacrylamide gels consisting of an 8% separation gel and 5% concentrated gel and subjected to electrophoresis at a constant 100 V. After electrophoresis, the gels were stained for 3 h using 0.07% (*w*/*v*) Coomassie Brilliant Blue R-250 in 30% (*v*/*v*) anhydrous ethanol and 7% (*v*/*v*) ice acetic acid. The bands were observed using a gel imaging system (BIO-RAD, Hercules, CA, USA).

### 2.5. Distribution of Type I and III Collagen in Intramuscular Connective Tissue (IMCT)

We applied indirect fluorescent immunostaining to determine the distribution of type I and III collagen in the IMCT. The method was based on Listrat [19] and Nakamura et al. [20] with some modifications. A frozen sectioning machine (SLEE medical GmbH, Nieder-Olm, Germany) was used to cut frozen sections of muscle at a temperature of −20 °C. Sections were attached to slides that had been precoated with polylysine and then fixed in 2% paraformaldehyde at 4 °C for 30 min. The sections were washed three times with phosphate-buffered saline (PBS), then incubated for 5 min at room temperature in 1% TritonX-100, followed by three PBS washes, and then sealed for 30 min at room temperature with 3% BSA. After these pretreatments, the sections were stained for collagen types I and III. Rabbit anti-sheep type 1 antibody (ab34710) was used for type I collagen staining and rabbit anti-bovine type III antibody (ab7778) was used for type III collagen staining, all diluted at 1:500 in PBS as the primary antibody and incubated overnight at 4 °C protected from light. After three washes using PBS, goat anti-rabbit IgG antibody, diluted at 1:200, was used as a secondary antibody and the sections were incubated for 60 min at 25 °C protected from light. Three more washes with PBS were applied and photographed under a fluorescent microscope (ECLIPSETi–U, Nikon, Tokyo, Japan).

### 2.6. Fourier Transform Infrared Spectroscopy (FTIR) Analysis of Type I and III Collagen

A 3 mg lyophilized collagen sample was accurately weighed and combined with 100 mg dry KBr. Then the sample was crushed with a mortar. A Fourier transform infrared spectrometer (Shimadzu, Kyoto, Japan) was used to detect the samples after pressing them into clear sheets using a powder tablet machine. Then the resulting spectra were analyzed using Origin software Version 9.6.5 [21].

### 2.7. Amino Acid Composition of Collagen Types I and III

An amino acid auto-analyzer was used to calculate the amounts of glycine (Gly), proline (Pro), alanine (Ala), serine (Ser), glutamic acid (Glu), arginine (Arg), threonine (Thr), valine (Val), histidine (His), leucine (Leu), aspartic acid (Asp), isoleucine (Ile), tyrosine (Tyr), phenylalanine (Phe), methionine (Met), lysine (Lys), and cystine (Cys) in type I and III collagen. The lyophilized collagen samples (100 mg) were hydrolyzed in 6 M HCl in a vacuum sealed tube at 110 °C for 24 h. The hydrolysate was filtered and diluted to a total volume of 50 mL, and then 1 mL was dried under reduced pressure and dissolved in 0.02 M HCl to prepare the samples. The analysis used a 4.6 × 60 mm column with #2622 resin. The column temperature was 57 °C and the reaction temperature was 135 °C. The buffer was citric acid–sodium citrate and the color development solution was ninhydrin.

### 2.8. Differential Scanning Calorimetry of Type I and III Collagen

The apparatus was preheated in vacuo for 30 min, then a 5 mg lyophilized collagen sample was weighed and compacted in an alumina crucible. Another alumina crucible was used as a control to start the heating test. The heating rate was 10 °C/min and high-purity nitrogen was used as the experimental atmosphere. The temperature range was from 25 °C to 75 °C [22].

### 2.9. Solubility Analysis of Type I and III Collagen

With reference to Hill [23] and Bergman [24], the solubility of collagen was calculated by measuring the hydroxyproline (Hyd) content. A 20 mg lyophilized collagen sample was weighed into a 10 mL centrifuge tube, 2 mL Green’s solution (potassium chloride 0.3 g, sodium chloride 8.6 g, and calcium chloride 0.28 g dissolved in distilled water and fixed to 1000 mL) was added and the sample was infiltrated at 4 °C overnight (12 h). The next day, the tubes were placed in a water bath at 77 °C for 63 min. After the water bath, the samples were cooled to room temperature and centrifuged at 4 °C and 1000× *g* for 30 min. Next, 1 mL Green’s solution was added to the precipitate and the centrifugation was repeated. The supernatant was transferred to a stoppered tube and an equal volume of concentrated HCl was added. The precipitate was transferred to another stoppered tube and 5 mL 6 M HCl was pipetted into the tube. The stoppered tube was sealed and hydrolyzed at 145 °C for 4 h. Afterwards, the acid was removed in a boiling water bath.

The contents of each tube were dissolved with 5 mL acetic acid–citric acid buffer (5.7 g sodium acetate–3H_2_O, 0.55 g citric acid, 3.75 g trisodium citrate-2H_2_O dissolved in 38.5 mL isopropanol and fixed to 100 mL with distilled water). After mixing, 1 mL solution was drawn, 2 mL isopropanol and 1 mL oxidant were added, and the resulting solution was shaken and mixed for 5 min at room temperature. Then 13 mL Ehrlich’s reagent solution (10 g p-dimethylaminobenzaldehyde dissolved in 15 mL 60% perchloric acid and mixed with isopropanol in a 3:13 volume ratio) was added and the mixture was heated in a water bath at 60 °C for 25 min. The supernatant and precipitation solution were fixed with isopropyl alcohol to 50 and 100 mL, respectively, and quantified colorimetrically at 558 nm. Finally, a standard curve was drawn to calculate the content of hydroxyproline.

The solubility of collagen was calculated as follows [25]:

Collagen content=hydroxyproline content × 7.46

$$\mathrm{Collagen}\;\mathrm{solubility}\;=\frac{\mathrm{collagen}\;\mathrm{content}\;\mathrm{in}\;\mathrm{supernatant}}{\mathrm{collagen}\;\mathrm{content}\;\mathrm{in}\;\left(\mathrm{supernatant}\;+\mathrm{precipitate}\right)}\times 100\%$$

### 2.10. Determination of Pyridinoline (Pyr) in IMCT

The Pyr in IMCT was determined by isolating the perimysium and endomysium in skeletal muscle as described in Light et al. [26]. A 100 g muscle sample was crushed and homogenized in 50 mM CaCl_2_ for 30 s and then filtered through a 1 mm^2^ round-well sieve. The material retained on the filter was homogenized again in 50 mM CaC1_2_ and re-filtered (repeated twice). The filtrate containing the endomysium was combined as the retained material. The filter residue containing perimysium was placed in 0.1% SDS, 5 mM tris-HC (pH 7.4) solution, centrifuged, and the precipitate was dialyzed in distilled water at 4 °C for 12 h to obtain the perimysium sample. The combined filtrate was mixed in 2.5 mM histidine, and 25 mM NaCl, 5 mM tris-HCl (pH 7.4) and then centrifuged (repeated 2–3 times); then, the precipitate was washed three times with 0.1% SDS, 5 mM tris-HC (pH 7.4) solution and dialyzed in distilled water at 4 °C for 12 h to obtain the endomysium samples. Finally, the endomysium and perimysium samples were freeze-dried for use.

A 100 mg lyophilized sample was weighed into a stoppered test tube and 8 mL 6 M HCl was added. The sample was hydrolyzed at 145 °C for 4 h. After hydrolysis, the acid was evaporated in a water bath at 100 °C, the residue was dissolved in 0.1 M HCl and centrifuged at 10,000× *g* for 20 min, and finally, the supernatant was freeze-dried.

Pyridinoline was subsequently determined by high-performance liquid chromatography (HPLC) on a Zorbax SB-C18 column (5 um, 4.6 × 250 mm, Agilent, Santa Clara, CA, USA) under the following conditions: fluorescence detection at Ex = 297 nm and Em = 390 nm; mobile phase A = 0.5% heptafluorobutyric acid (HFBA) and mobile phase B = acetonitrile; and flow rate = 1 mL/min. The lyophilized sample was dissolved in 200 mL 0.5% HFBA, diluted to a suitable volume, and 10 mL was used as the sample for analysis.

### 2.11. Statistical Analysis

All experiment data are presented as mean ± standard deviation. The experimental data were subjected to analysis of variance (ANOVA) using SPSS 16.0 software. Duncan’s multiple range test was used to assess the differences between variables at the 5% level of significance. Differences were considered significant at *p* < 0.05. Pearson’s correlation coefficient was used to evaluate the relationships between collagen characteristics.

## 3. Results and Discussion

### 3.1. Expression Analysis of the COL1A1, COL1A2, and COL3A1 Genes

**Figure 1:**
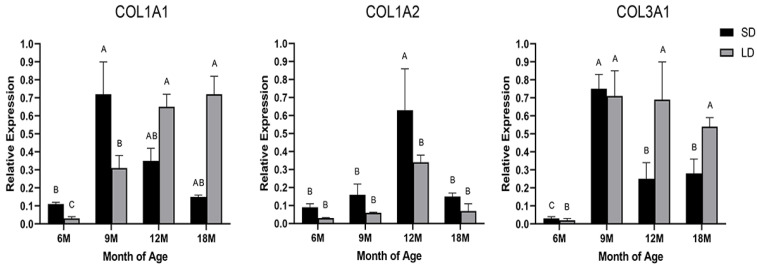
The mRNA abundance of (**COL1A1**, **COL1A2**), and (**COL3A1**) in the semitendinosus (SD) and longissimus dorsi (LD) muscles at different growth stages. Different capital letters indicate significant differences between different months in the same muscle tissue.

Type I and III collagens are the main fibrous collagens that make up the connective tissue of skeletal muscle and participate in the biosynthesis of collagen fibrous tissue and collagen. The synthesis rates of type I and III collagens are regulated by their corresponding mRNA expression levels [27]. In this study, COL1A1 expression in the SD muscle peaked at 9 months of age and thereafter decreased with the age of the *Wuzhumuqin sheep*, whereas expression in the LD muscle increased with age (Figure 1). COL1A2 expression increased as the various muscles developed, reaching an apex at 12 months of age, and then it decreased. In addition, the SD showed a higher level of expression than the LD muscle. Previous studies of beef samples have found that tender meat contains less collagen α1 and α2 than tough meat, which is associated with decreased shear values [28]. Furthermore, cellular investigations have found that COL1A1 is closely related to myoblast proliferation [29]. On the other hand, COL3A1 expression peaked at 9 months of age throughout the different stages of muscle growth, and it decreased to various degrees with age. COL3A1 mRNA abundance was higher in the skeletal muscle of cull-cows (74.9 ± 3.2 months age) than in heifers (18.4 ± 3.2 months age) fed high-energy diets [30]. However, in another study, a lower mRNA expression of COL3A1 was found in 30-month-old heifers compared to heifers at 20 and 25 months of age [31]. COL3A1 mRNA expression was positively connected with the collagen content and insoluble collagen content in pig skeletal muscle connective tissue at different growth stages, while it was negatively correlated with collagen solubility [32]. However, there was no correlation between the expression of COL3A1 and collagen content and solubility in the longissimus thoracis of cattle [30]. These previous studies showed that COL3A1 is expressed and correlated variously in the muscle tissue of various species and ages. At the same time, the potential impacts of elevated COL3A1 expression on meat quality cannot be disregarded [33]. In the current study, collagen-related genes were highly expressed in the muscles of *Wuzhumuqin sheep* at 9 and 12 months of age, and may therefore be essential for IMCT at that age.

### 3.2. Distribution of Type I and III Collagen in Intramuscular Connective Tissue

**Figure 2:**
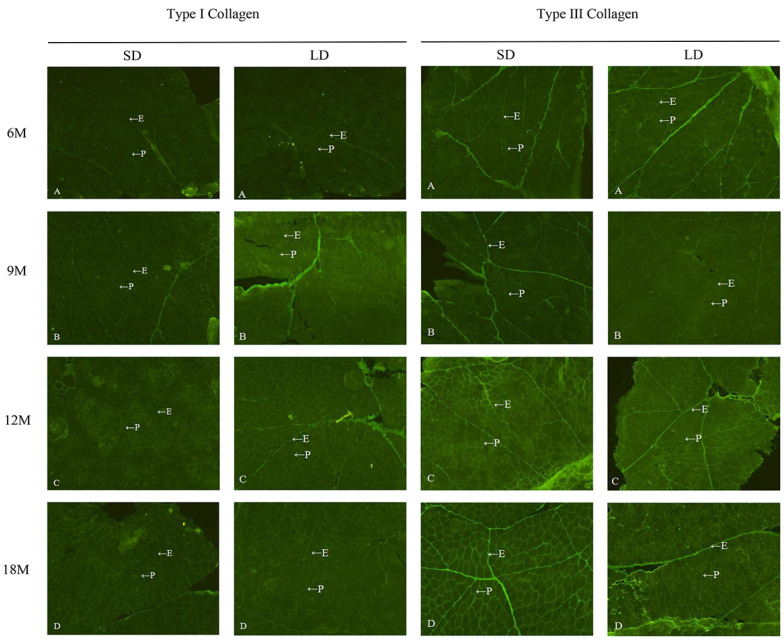
Distribution of type (**Ⅰ**, **Ⅲ**) collagen in semitendinosus (SD) and longissimus dorsi (LD) muscles at different growth stages. The endomysium is represented by E, perimysium by P, and (**A**–**D**) represent 6 (6 M), 9 (9 M), 12 (12 M), and 18 (18 M) months of age, respectively. Magnification is 10×.

The distribution of collagen types I and III in the connective tissues of the LD and SD muscles of *Wuzhumuqin sheep* at various ages were investigated by immunofluorescence staining (Figure 2). Both were primarily distributed in the endomysium and perimysium of the LD and SD muscles in the IMCT, in line with a previous study on bovine muscle [34]. Normally, the composition and amount of IMCT in domestic animals are influenced by conditions such as muscle location and age [20]. In the current study, type I and type III collagen gradually became more visible in connective tissue as sheep aged. Compared to type I collagen, type III was more visible in the endomysium and perimysium. This may suggest that the content of each type increases with aging but that the level of type III is higher than that of type I. However, no significant differences were found in the different muscles, and further confirmation is required by considering the Hyd content of collagen [35].

### 3.3. Isolation and Identification of Type I and III Collagen

**Figure 3:**
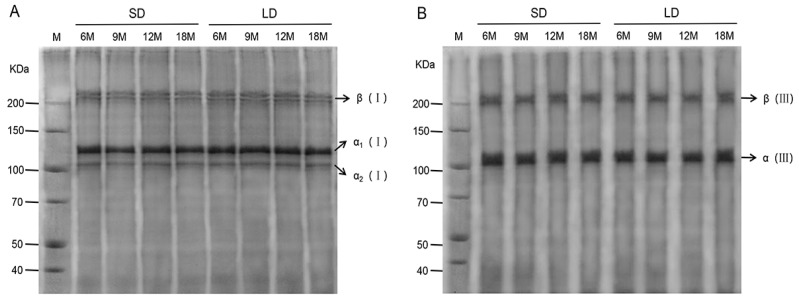
SDS-PAGE analysis of type Ⅰ collagen (**A**) and type Ⅲ collagen (**B**) from the semitendinosus (SD) and longissimus dorsi (LD) muscles at different growth stages. The first lane is the Marker (M).

The SDS-PAGE analysis of collagen isolated from the SD and LD muscle at 6, 9, 12, and 18 months of age were shown in Figure 3. At each age, the target bands of the SD and LD samples were visible, and there was little variation in the color of the bands at the same molecular weight. In type I collagen, two single peptide chains of α1 (I) and α2 (I) appeared at about 100 KDa. They were clearly separated from their dimers β (I) near 210 KDa, similar to the findings of Liu et al. [36]. The results for type III collagen were similar. The dimers β (III) and α (III) of type III collagen were likewise efficiently separated. Compared to the Marker (M), an α (III) single peptide chain appeared at about 100 KDa, and the dimer appeared near 200 KDa β (III). Therefore, the collagen isolated from the muscle was classified as type I and type III collagen. Furthermore, no random bands were noted, indicating that the extracted materials were of excellent purity and that the collagen was not degraded into small molecular peptides during the extraction process, providing a solid foundation for further research.

**Figure 4:**
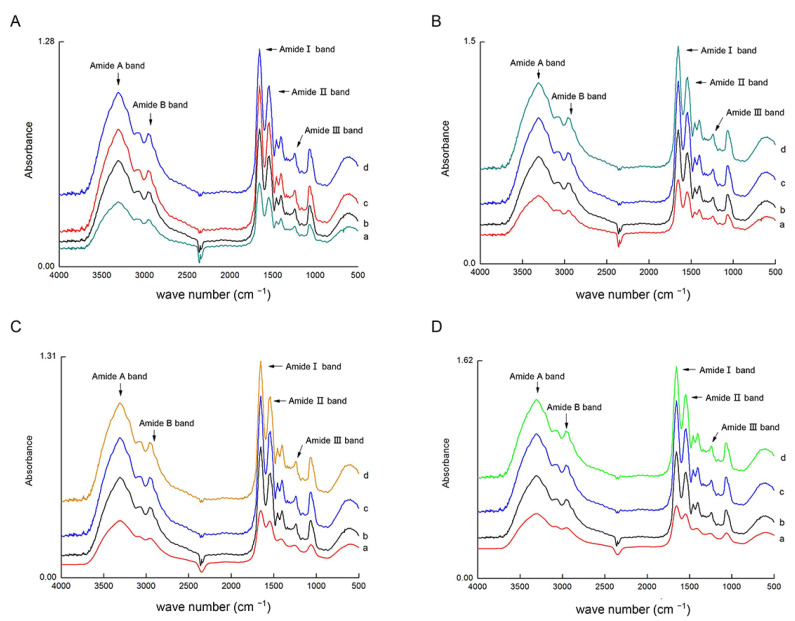
FTIR spectra of type Ⅰ and Ⅲ collagen from the semitendinosus (SD) and longissimus dorsi (LD) muscles at different growth stages. (**A**,**B**) represent type Ⅰ collagen from the SD and LD; (**C**,**D**) represent type Ⅲ collagen from the SD and LD; and a, b, c, and d represent 6, 9, 12, and 18 months of age, respectively.

Furthermore, we investigated the secondary structure of type I and type III collagen via FTIR (Figure 4). The main absorption peaks appeared in all collagen samples, and the peak forms were similar, but the wave frequencies of the same absorption peaks were slightly different. The absorption properties of the amide A band were closely related to the N-H stretching vibration. Generally, the free N-H stretching vibration takes place in the range of 3400–3440 cm^−1^. When N-H functional groups are coupled with hydrogen bonds, the energy band shifts to a lower frequency, usually around 3300 cm^−1^ [37]. At 6, 9, 12, and 18 months of age, the wave frequencies of the amide A band absorption peaks of type I and type III collagen were 3308, 3307, 3303, and 3300 cm^−1^, respectively, with the wave frequencies decreasing with age. This suggests that the N-H group of connective tissue collagen in sheep muscle has a high concentration of hydrogen bonds, in line with findings in sheep bone collagen [38]. The amide B band is an asymmetric CH2 vibrational peak that appears in all collagen samples near the wave frequency of 2945 cm^−1^. The amide I band, a C=O stretching vibrational peak with a characteristic frequency range of 1600–1700 cm^−1^, is sensitive to changes in secondary structure in collagen and therefore is frequently used as a functional group to define collagen secondary structure integrity [39]. All collagen samples had intact amide I band peaks near 1652 cm^−1^. The amide II band is a stretched vibrational peak formed by the combination of NH and CN, and absorption peaks may be detected in all collagen samples around 1550 cm^−1^. The amide III band at 1240 cm^−1^ is a vibrational peak formed in each collagen sample by the combination of NH and CN. The ratios of the absorbance value between each peak of the amide III band and 1454 cm^−1^ ranged from 0.90 to 0.93, close to 1.0, indicating that the triple helix structure of skeletal muscle intramuscular collagen was mostly intact [40].

### 3.4. Amino Acid Composition of Type I and III Collagen

After the collagen had fully hydrolyzed, 17 amino acids in type I and type III collagen of the intramuscular connective tissue of *Wuzhumuqin sheep* at different months of age were detected using an amino acid analyzer. Spectrophotometric techniques were used to determine the Hyd content (Table 1). We found that the LD muscle had a higher overall collagen type I and III amino acid content than the SD muscle. In the different growth stages, the total amino acid content of type I collagen in muscle increased significantly with age, whereas type III collagen had the opposite result. Glycine was the main constituent amino acid in collagen, accounting for 33.2% to 34.4% of the total amino acid content, depending on the collagen source [41]. In our study, Gly made up the largest part of all amino acids, which accounts for about 1/3 of the total content. Its content in different muscles and the pattern of variation between ages was similar to that of the total amino acids. In addition, large amounts of Glu and alanine, Pro, and Hyd, as well as small amounts of Cys, Tyr, and Met, were found, consistent with previous research [42]. Therefore, the purity of the isolated collagen was verified from the perspective of the amino acid composition. Hydroxyproline is a unique amino acid found in collagen that can be used to assess collagen quantity [35]. It increases with age in both type I and type III collagen isolated from *Wuzhumuqin sheep* skeletal muscle, and, in turn, the collagen content gradually increases with age. In general, imino acids (Hyd and Pro) stabilize collagen by preserving the integrity of the triple helix structure [43]. The pyrrolidine rings of Pro and Hyp constrain the conformation of the polypeptide chain in the secondary structure and contribute to the strengthening of the triple helix. Hydroxyproline also helps the stabilization of the triple helix structure by forming interchain hydrogen bonds through hydroxyl groups and contributes to the thermal stability of collagen [37]. The levels of imino acids increased significantly with age in type I collagen, but increased significantly from 6 to 12 months and then decreased significantly from 12 to 18 months in type III collagen. This was mainly due to the Pro content. These results indicate that collagen stability in the IMCT of sheep increases with age.

### 3.5. Collagen Solubility Analysis

The solubility of type I collagen in the skeletal muscles of *Wuzhumuqin sheep* decreased significantly with age (Table 2). Similarly, type III collagen solubility decreased significantly with age in the LD muscle, while it tended to decrease with age in the SD muscle but was only significant between 6 months of age and the other months of age. At the same age, solubility was significantly higher for type I than for type III. The reductive covalent cross-linking of collagen gradually switches to stable non-reductive cross-linking as animals age. The stabilization of cross-linking decreases collagen solubility and increases mechanical strength, resulting in less-tender meat [44]. As animals age, collagen molecules in the connective tissue of the muscle become increasingly cross-linked, creating a tighter spatial network structure and thereby slowing the process of protein hydrolysis. Thus, younger muscles in *Wuzhumuqin sheep* may have greater tenderness.

### 3.6. Analysis of the Denaturation Temperature of Collagen Types I and III

**Figure 5:**
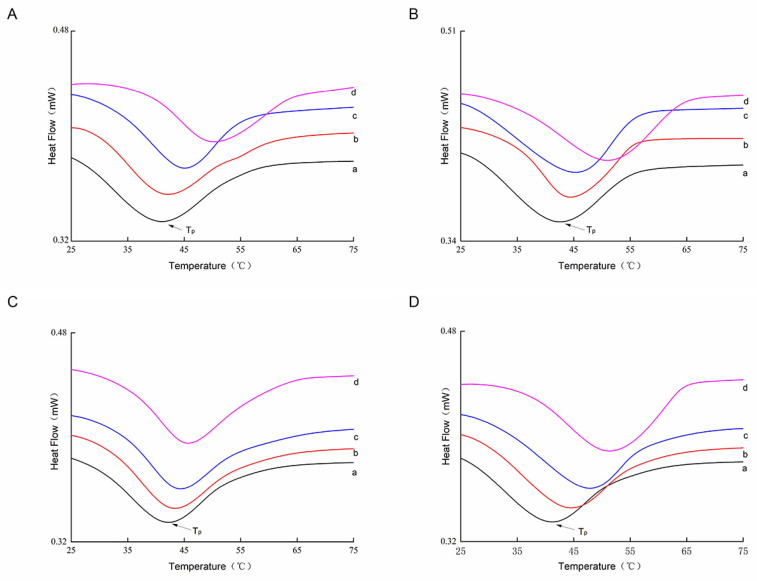
DSC analysis of type Ⅰ and Ⅲ collagen in the semitendinosus (SD) and longissimus dorsi (LD) muscles in different growth stages. (**A**,**B**) represent type Ⅰ collagen from the SD and LD; (**C**,**D**) represent type Ⅲ collagen from the SD and LD; and a, b, c, and d represent 6, 9, 12, and 18 months of age, respectively.

DSC heat flow analysis profiles at different months of age are shown in Figure 5 and Table 3. The highest peak temperature, T_P_, is typically used to indicate the thermal denaturation temperature of a collagen sample [45]. Significant heat absorption peaks appeared in all curves, and both types of collagen displayed a consistent trend in which the three denaturation temperatures (T_o_, T_P_, and T_f_) increased with age. The degree of covalent cross-links of collagen in muscle increases with age; at the same time, the thermal stability of collagen increases and solubility decreases [46]. Thus, as the *Wuzhumuqin sheep* aged, the unstable immature cross-links in the muscle collagen gradually formed into stable mature cross-links, leading to solid collagen molecules and resulting in increased thermal stability. In addition, the imino acid content of collagen is positively associated with thermal stability [43]. In our study, the higher imino acid content resulted in better thermal stability of the collagen in older sheep. This result concurs with the findings of collagen solubility.

### 3.7. Collagen Cross-Linking Content Analysis

We analyzed the content of collagen cross-linking by measuring Pyr via HPLC (Table 4). With the exception of the nonsignificant difference between 6 and 9 months of age in perimysium, the amount of cross-linked collagen Pyr in the perimysium and the endomysium of the skeletal muscle of *Wuzhumuqin sheep* increased significantly with age. The endomysium has a substantially higher Pyr content than the perimysium, and thus the endomysium differential is crucial in terms of the influence of IMCT on meat quality [47]. For samples of the same age, the Pyr content in the IMCT of the SD was higher than that in the LD, consistent with Bosselmann et al. [11]. The Pyr content was low in young animals because the majority of the collagen cross-links were reducible Schiff bases, which are sensitive to acid and heat. However, as the animal ages, the covalent bonds between the collagen molecules are lost, resulting in the formation of non-reducible mature cross-links. Non-reducible collagen cross-linking Pyr is thought to play a key role in this process. The amount of Pyr cross-links in muscle changes with the total collagen content and is related to decreased collagen solubility and increased meat toughness as the animal ages [48]. Previous studies have found a relationship between collagen cross-linking, solubility, thermal stability, and amino acid content. Thus, their relationships may have significant implications for IMCT collagen. However, the relationships between the collagen-related characteristics in the IMCT of *Wuzhumuqin sheep* are currently unknown and need to be clarified via correlation analysis.

### 3.8. Correlation Analysis of Collagen Characteristics

**Figure 6:**
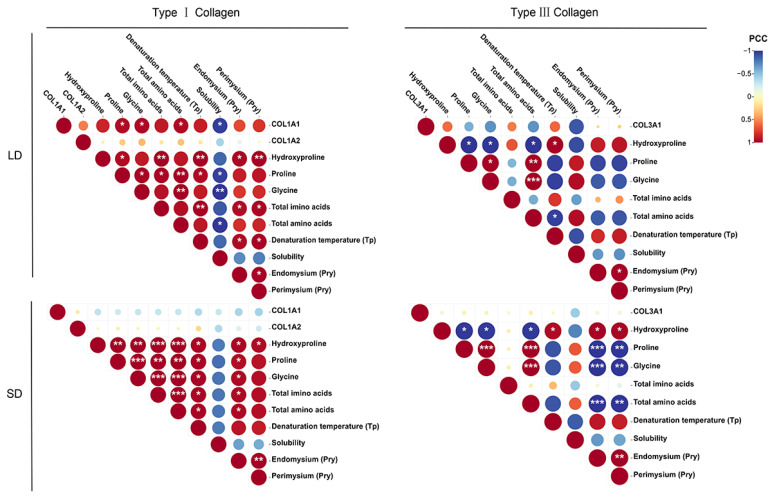
Correlation analysis of the collagen characteristics. The size and color of the circles indicate the strength of the correlations. PCC: Pearson correlation coefficient. * *p* < 0.05, ** *p* < 0.01, *** *p* < 0.001.

Correlation analyses were performed to investigate the relationships among the collagen characteristics, including mRNA expression, amino acid content, denaturation temperature (T_p_), solubility, and cross-linking content (Pry). As shown in Figure 6, most of the characteristics were positively correlated. Only solubility was negatively correlated with other variables, particularly in type I collagen in LD, where it was significantly negatively correlated with COL1A1 gene expression and the total amino acid content (*p* < 0.05). In SD, there was a weak correlation between collagen characteristics and collagen-related genes, while COL1A1 gene expression in LD was significantly positively correlated with the Pro, Gly, and total amino acid levels (*p* < 0.05). Previous studies have yielded inconsistent results regarding the correlation between COL3A1 gene expression and collagen characteristics [30,32]. By contrast, the expression of the COL1A1 gene was positively correlated with the collagen characteristics of LD, suggesting that this gene may be a crucial biomarker in the IMCT of LD. In a previous study, the amino acids in collagen were mainly Gly and Pro, while Hyd was the signature amino acid of collagen [49]. In the present study, a positive correlation was mainly observed between these three amino acids, with a highly significant positive correlation observed in SD type I collagen (*p* < 0.01). However, in type III collagen, the Hyd content had a significant negative correlation with the Pro, Gly, and total amino acid contents (*p* < 0.05). The relationships between amino acids in collagen may therefore depend on collagen type. The collagen denaturation temperature (T_p_) and amino acid content also had a significant positive correlation in type I collagen (*p* < 0.05), with a highly significant positive correlation in LD with Hyp and imino acids (*p* < 0.01). However, only Hyd had a significant positive correlation with denaturation temperature in type III collagen (*p* < 0.05). This indicates that an increase in imino acid with age leads to the increased thermal stability of IMCT collagen; the same result has been observed in bone collagen from sheep [38]. In addition, the degree of IMCT cross-linking was also strongly correlated with the amino acid content. Hydroxyproline had a significant positive correlation with the Pry content in LD and SD type I collagen and SD type III collagen (*p* < 0.05). Furthermore, the endomysium Pry content in SD type I collagen had a significant positive correlation with the levels of Pro, Gly, total imino acids, and total amino acids (*p* < 0.05). By contrast, there was little correlation between the Pry content and amino acids in LD type III collagen (*p* > 0.05). The Pry content in SD muscle type III collagen had a strong significant negative correlation with the levels of Pro, Gly, and total amino acids (*p* < 0.01), while the endomysium Pry content was more strongly negatively correlated with these amino acids (*p* < 0.001). The strong significant correlation between the endomysium Pry content and amino acid content in SD may explain the higher degree of cross-linking in SD. These results indicated various correlations between collagen characteristics. We further hypothesize that the correlation between collagen characteristics may affect meat quality parameters. Researchers have found that soluble collagen was negatively correlated with shear force in sheep biceps femoris [50,51]. Additionally, type III collagen had a negative correlation with pig and bovine muscle tenderness [9,13]. In this study, we investigated the characteristics of type I and type III collagen in naturally grazed sheep muscle at different growth stages and elucidated the relationship between them. However, the effect of type I and type III collagen on sheep meat quality parameters needs to be investigated further.

## 4. Conclusions

The muscle tissue had the highest expression of collagen-related genes at 9 and 12 months of age. We isolated and classified type I and III collagens with a stable triple helix structure and found that they were widely dispersed in the endomysium and perimysium. The amino acid content, solubility, thermal stability, and degree of cross-linking of sheep IMCT collagen vary depending on the type of collagen, muscle type, and age. Furthermore, the correlation analysis highlighted multiple significant correlations between collagen characteristics and suggested that COL1A1 could be a crucial biomarker in the IMCT of LD. These findings could provide the theoretical basis for future research on the relationships between IMCT collagen and meat quality.

## Figures and Tables

**Table 1:** Amino acid composition of type Ⅰ and Ⅲ collagen from Semitendinosus (SD) and Longissimus dorsi (LD) in different growth stages.

Amino Acids	Muscles	Type I Collagen				Type III Collagen			
		6M	9M	12M	18M	6M	9M	12M	18M
Aspartic acid	SD	0.24 ± 0.02 ^D^	1.61 ± 0.12 ^C^	2.72 ± 0.18 ^B^	4.14 ± 0.32 ^A^	4.46 ± 0.32 ^D^	3.48 ± 0.23 ^C^	3.27 ± 0.19 ^B^	1.86 ± 0.12 ^A^
	LD	4.25 ± 0.21 ^D^	5.09 ± 0.26 ^C^	5.52 ± 0.31 ^B^	6.09 ± 0.32 ^A^	6.09 ± 0.22 ^D^	5.31 ± 0.21 ^C^	5.18 ± 0.18 ^B^	4.63 ± 0.15 ^A^
Threonine	SD	0.57 ± 0.06 ^D^	0.61 ± 0.07 ^C^	1.00 ± 0.09 ^B^	3.79 ± 0.34 ^A^	2.60 ± 0.21 ^B^	2.66 ± 0.22 ^C^	2.57 ± 0.18 ^B^	1.08 ± 0.09 ^A^
	LD	3.02 ± 0.11 ^D^	3.97 ± 0.13 ^C^	3.47 ± 0.16 ^B^	3.67 ± 0.21 ^A^	3.09 ± 0.14 ^D^	2.21 ± 0.11 ^C^	2.09 ± 0.13 ^B^	1.53 ± 0.08 ^A^
Serine	SD	0.51 ± 0.05 ^D^	0.85 ± 0.08 ^C^	1.32 ± 0.12 ^B^	2.40 ± 0.21 ^A^	2.36 ± 0.22 ^C^	2.44 ± 0.23 ^B^	2.38 ± 0.21 ^B^	1.68 ± 0.15 ^A^
	LD	2.85 ± 0.09 ^C^	3.22 ± 0.13 ^B^	3.74 ± 0.12 ^A^	2.81 ± 0.11 ^C^	2.95 ± 0.17 ^D^	2.37 ± 0.15 ^C^	2.25 ± 0.12 ^B^	1.95 ± 0.10 ^A^
Glutamic acid	SD	1.30 ± 0.12 ^D^	1.84 ± 0.16 ^C^	2.99 ± 0.26 ^B^	6.97 ± 0.54 ^A^	6.8 ± 0.51 ^D^	5.17 ± 0.43 ^C^	4.58 ± 0.36 ^B^	2.45 ± 0.16 ^A^
	LD	4.62 ± 0.22 ^D^	5.25 ± 0.26 ^C^	6.05 ± 0.18 ^B^	6.72 ± 0.27 ^A^	6.44 ± 0.31 ^D^	5.12 ± 0.26 ^C^	4.66 ± 0.15 ^B^	3.34 ± 0.12 ^A^
Glycine	SD	4.55 ± 0.43 ^D^	8.44 ± 0.61 ^C^	13.16 ± 0.98 ^B^	26.61 ± 1.14 ^A^	23.79 ± 1.61 ^D^	23.37 ± 1.54 ^C^	23.03 ± 1.22 ^B^	16.89 ± 0.86 ^A^
	LD	18.21 ± 0.82 ^D^	23.96 ± 0.63 ^C^	26.25 ± 0.71 ^B^	29.31 ± 0.46 ^A^	27.33 ± 0.81 ^D^	23.66 ± 0.62 ^C^	23.18 ± 0.46 ^B^	19.86 ± 0.37 ^A^
Alanine	SD	1.29 ± 0.11 ^D^	2.51 ± 0.22 ^C^	3.63 ± 0.31 ^B^	7.21 ± 0.64 ^A^	6.5 ± 0.32 ^C^	6.78 ± 0.36 ^B^	6.79 ± 0.34 ^B^	4.44 ± 0.19 ^A^
	LD	5.41 ± 0.21 ^D^	7.05 ± 0.24 ^C^	7.73 ± 0.13 ^B^	8.62 ± 0.18 ^A^	7.93 ± 0.41 ^D^	6.70 ± 0.36 ^C^	6.58 ± 0.34 ^B^	5.58 ± 0.31 ^A^
Cystine	SD	0.01 ± 0 ^B^	0.01 ± 0 ^B^	0.02 ± 0 ^B^	0.02 ± 0 ^A^	0.01 ± 0 ^C^	0.07 ± 0 ^B^	0.04 ± 0 ^A^	0.05 ± 0 ^A^
	LD	0.09 ± 0 ^B^	0.07 ± 0 ^B^	0.08 ± 0 ^B^	0.03 ± 0 ^A^	0.04 ± 0 ^C^	0.05 ± 0 ^B^	0.03 ± 0 ^A^	0.03 ± 0 ^A^
Valine	SD	0.52 ± 0.05 ^D^	0.71 ± 0.07 ^C^	0.98 ± 0.08 ^B^	1.49 ± 0.13 ^A^	1.80 ± 0.14 ^C^	1.77 ± 0.13 ^C^	1.73 ± 0.11 ^B^	0.93 ± 0.06 ^A^
	LD	2.26 ± 0.08 ^C^	3.26 ± 0.12 ^A^	2.27 ± 0.13 ^BC^	2.36 ± 0.11 ^B^	1.91 ± 0.12 ^D^	1.74 ± 0.11 ^C^	1.65 ± 0.02 ^B^	1.28 ± 0.07 ^A^
Methionine	SD	0.14 ± 0.01 ^C^	0.17 ± 0.02 ^BC^	0.18 ± 0.02 ^B^	0.76 ± 0.06 ^A^	0.14 ± 0.01 ^D^	0.43 ± 0.03 ^C^	0.47 ± 0.04 ^B^	0.70 ± 0.06 ^A^
	LD	0.32 ± 0.01 ^D^	0.52 ± 0.02 ^C^	0.63 ± 0.02 ^B^	0.99 ± 0.04 ^A^	0.63 ± 0.03 ^D^	0.53 ± 0.03 ^C^	0.49 ± 0.02 ^B^	0.28 ± 0.02 ^A^
Isoleucine	SD	0.30 ± 0.03 ^D^	0.41 ± 0.04 ^C^	0.53 ± 0.04 ^B^	0.79 ± 0.06 ^A^	0.23 ± 0.02 ^D^	0.19 ± 0.02 ^C^	0.09 ± 0.01 ^B^	0.42 ± 0.03 ^A^
	LD	0.29 ± 0.01 ^D^	0.23 ± 0.01 ^C^	0.34 ± 0.02 ^B^	0.55 ± 0.01 ^A^	0.69 ± 0.04 ^D^	0.47 ± 0.03 ^C^	0.37 ± 0.03 ^B^	0.22 ± 0.01 ^A^
Leucine	SD	0.13 ± 0.01 ^D^	0.21 ± 0.02 ^C^	0.42 ± 0.04 ^B^	0.92 ± 0.08 ^A^	0.52 ± 0.05 ^C^	0.56 ± 0.05 ^C^	0.29 ± 0.24 ^B^	0.74 ± 0.053 ^A^
	LD	0.28 ± 0.01 ^D^	0.36 ± 0.02 ^C^	0.41 ± 0.02 ^B^	0.45 ± 0.03 ^A^	0.49 ± 0.03 ^C^	0.41 ± 0.02 ^B^	0.32 ± 0.02 ^A^	0.30 ± 0.02 ^A^
Tyrosine	SD	0.03 ± 0 ^A^	0.02 ± 0 ^A^	0.04 ± 0 ^A^	0.02 ± 0 ^A^	0.10 ± 0 ^A^	0.09 ± 0 ^A^	0.04 ± 0 ^C^	0.09 ± 0 ^A^
	LD	0.04 ± 0 ^D^	0.03 ± 0 ^C^	0.08 ± 0 ^B^	0.06 ± 0 ^A^	0.06 ± 0 ^C^	0.04 ± 0 ^B^	0.04 ± 0 ^B^	0.03 ± 0 ^AB^
Phenylalanine	SD	0.37 ± 0.04 ^D^	0.56 ± 0.05 ^C^	0.88 ± 0.07 ^B^	1.28 ± 0.11 ^A^	1.48 ± 0.12 ^C^	1.45 ± 0.11 ^C^	1.41 ± 0.09 ^B^	1.04 ± 0.08 ^A^
	LD	1.73 ± 0.08 ^C^	1.47 ± 0.09 ^B^	1.46 ± 0.10 ^B^	1.28 ± 0.08 ^A^	1.94 ± 0.14 ^D^	1.55 ± 0.12 ^C^	1.50 ± 0.11 ^B^	1.08 ± 0.08 ^A^
Lysine	SD	0.43 ± 0.04 ^D^	0.73 ± 0.06 ^C^	1.02 ± 0.10 ^B^	2.35 ± 0.21 ^A^	2.11 ± 0.16 ^D^	5.09 ± 0.37 ^C^	5.03 ± 0.29 ^B^	1.49 ± 0.12 ^A^
	LD	1.43 ± 0.08 ^D^	3.26 ± 0.11 ^C^	3.55 ± 0.16 ^B^	3.76 ± 0.12 ^A^	4.40 ± 0.36 ^D^	3.30 ± 0.27 ^C^	3.14 ± 0.22 ^B^	2.64 ± 0.14 ^A^
Histidine	SD	0.12 ± 0.01 ^D^	0.21 ± 0.02 ^C^	0.32 ± 0.03 ^B^	0.98 ± 0.07 ^A^	0.68 ± 0.07 ^C^	0.39 ± 0.03 ^B^	0.54 ± 0.04 ^A^	0.55 ± 0.05 ^A^
	LD	0.16 ± 0.01 ^D^	0.26 ± 0.01 ^C^	0.34 ± 0.02 ^B^	0.47 ± 0.03 ^A^	0.35 ± 0.02 ^C^	0.34 ± 0.02 ^C^	0.32 ± 0.01 ^B^	0.20 ± 0.01 ^A^
Arginine	SD	0.66 ± 0.06 ^D^	1.00 ± 0.09 ^C^	1.61 ± 0.12 ^B^	2.12 ± 0.16 ^A^	6.14 ± 0.51 ^D^	5.03 ± 0.46 ^C^	4.91 ± 0.41 ^B^	3.99 ± 0.21 ^A^
	LD	2.31 ± 0.09 ^D^	4.52 ± 0.21 ^C^	5.13 ± 0.14 ^B^	6.02 ± 0.24 ^A^	5.95 ± 0.32 ^D^	5.47 ± 0.31 ^C^	5.38 ± 0.27 ^B^	4.45 ± 0.19 ^A^
Hydroxyproline	SD	1.75 ± 0.16 ^D^	3.3 ± 0.22 ^C^	5.22 ± 0.43 ^B^	11.72 ± 1.03 ^A^	5.51 ± 0.34 ^D^	5.69 ± 0.36 ^C^	6.14 ± 0.42 ^B^	7.78 ± 0.51 ^A^
	LD	1.48 ± 0.07 ^D^	2.58 ± 0.14 ^C^	3.98 ± 0.17 ^B^	8.01 ± 0.43 ^A^	5.02 ± 0.21 ^D^	5.91 ± 0.31 ^C^	6.56 ± 0.27 ^B^	7.53 ± 0.23 ^A^
Proline	SD	1.4 ± 0.12 ^D^	2.65 ± 0.21 ^C^	4.08 ± 0.28 ^B^	7.66 ± 0.44 ^A^	7.28 ± 0.67 ^D^	7.16 ± 0.55 ^C^	6.99 ± 0.48 ^B^	5.09 ± 0.29 ^A^
	LD	6.25 ± 0.31 ^D^	7.38 ± 0.28 ^C^	8.13 ± 0.34 ^B^	9.3 ± 0.42 ^A^	8.03 ± 0.46 ^D^	7.19 ± 0.39 ^C^	6.99 ± 0.37 ^B^	5.81 ± 0.28 ^A^
Total imino acids	SD	3.15 ± 0.26 ^D^	5.95 ± 0.41 ^C^	9.30 ± 0.53 ^B^	19.38 ± 0.32 ^A^	12.79 ± 0.41 ^C^	12.85 ± 0.39 ^A^	13.13 ± 0.31 ^B^	12.87 ± 0.33 ^A^
	LD	7.73 ± 0.41 ^D^	9.96 ± 0.37 ^C^	12.11 ± 0.64 ^B^	17.31 ± 0.71 ^A^	13.05 ± 0.39 ^D^	13.1 ± 0.51 ^C^	13.55 ± 0.48 ^B^	13.34 ± 0.25 ^A^
Total amino acids	SD	14.32 ± 0.36 ^D^	25.84 ± 0.46 ^C^	40.12 ± 0.63 ^B^	81.23 ± 0.96 ^A^	72.51 ± 0.63 ^D^	71.82 ± 0.78 ^C^	70.3 ± 0.46 ^B^	51.27 ± 0.28 ^A^
	LD	55.00 ± 0.72 ^D^	72.48 ± 0.91 ^C^	79.16 ± 0.67 ^B^	90.5 ± 0.74 ^A^	83.34 ± 0.44 ^D^	72.37 ± 0.38 ^C^	70.73 ± 0.31 ^B^	60.74 ± 0.29 ^A^

Data are presented as mean ± standard deviation. Different capital letters in the same row indicate significant differences (*p* < 0.05).

**Table 2:** The percentage of type Ⅰ and Ⅲ collagen solubility from Semitendinosus (SD) and Longissimus dorsi (LD) in different growth stages.

		6M	9M	12M	18M
SD	Type Ⅰ Collagen	15.24 ± 0.43 ^aA^	10.63 ± 0.47 ^bA^	9.96 ± 0.42 ^cA^	8.72 ± 0.31 ^dA^
	Type Ⅲ Collagen	13.89 ± 0.62 ^aB^	10.02 ± 0.43 ^bB^	9.41 ± 0.34 ^bcB^	8.14 ± 0.32 ^cB^
LD	Type Ⅰ Collagen	16.01 ± 0.54 ^aA^	11.82 ± 0.51 ^bA^	10.46 ± 0.39 ^cA^	9.12 ± 0.29 ^dA^
	Type Ⅲ Collagen	14.62 ± 0.52 ^aB^	10.21 ± 0.43 ^bB^	9.85 ± 0.41 ^cB^	8.63 ± 0.36 ^dB^

Data are presented as mean ± standard deviation. Different capital letters in the same row indicate significant differences (*p* < 0.05), and different lowercase letters indicate significant differences between different collagen in the same muscle (*p* < 0.05).

**Table 3:** DSC analysis of type Ⅰ and Ⅲ collagen in Semitendinosus (SD) and Longissimus dorsi (LD) in different growth stages.

Denaturation Temperature			6M	9M	12M	18M
SD	Type Ⅰ Collagen	T_o_ (°C)	30.5 ± 0.45 ^A^	32.7 ± 0.37 ^B^	36.3 ± 0.21 ^C^	41.7 ± 0.26 ^D^
		T_p_ (°C)	40.7 ± 0.70 ^A^	41.3 ± 0.62 ^B^	44.8 ± 0.42 ^C^	48.7 ± 0.48 ^D^
		T_f_ (°C)	49.7 ± 0.38 ^A^	50.5 ± 0.71 ^B^	51.4 ± 0.56 ^C^	60.5 ± 0.82 ^D^
	Type Ⅲ Collagen	T_o_ (°C)	33.1 ± 0.51 ^A^	35.1 ± 0.45 ^B^	36.9 ± 0.54 ^C^	38.4 ± 0.48 ^D^
		T_p_ (°C)	41.9 ± 0.46 ^A^	42.6 ± 0.73 ^B^	43.6 ± 0.62 ^C^	45.0 ± 0.57 ^D^
		T_f_ (°C)	49.7 ± 0.62 ^A^	50.7 ± 0.61 ^B^	51.2 ± 0.73 ^C^	52.4 ± 0.69 ^D^
LD	Type Ⅰ Collagen	T_o_ (°C)	31.6 ± 0.37 ^A^	37.9 ± 0.27 ^B^	32.1 ± 0.31 ^C^	39.3 ± 0.31 ^D^
		T_p_ (°C)	42.1 ± 0.43 ^A^	43.1 ± 0.31 ^B^	45.8 ± 0.36 ^C^	50.7 ± 0.38 ^D^
		T_f_ (°C)	50.7 ± 0.58 ^A^	52.4 ± 0.29 ^B^	51.8 ± 0.35 ^C^	59.1 ± 0.61 ^D^
	Type Ⅲ Collagen	T_o_ (°C)	30.4 ± 0.36 ^A^	33.3 ± 0.49 ^B^	35.9 ± 0.27 ^C^	40.1 ± 0.42 ^D^
		T_p_ (°C)	41.2 ± 0.39 ^A^	44.3 ± 0.51 ^B^	48.1 ± 0.39 ^C^	50.6 ± 0.47 ^D^
		T_f_ (°C)	48.7 ± 0.41 ^A^	52.1 ± 0.68 ^B^	54.4 ± 0.48 ^C^	61.1 ± 0.64 ^D^

T_o_: onset temperature, T_p_: peak temperature, T_f_: final temperature. Data are presented as mean ± standard deviation, and different capital letters in the same row indicate significant differences (*p* < 0.05).

**Table 4:** The content of pyridinoline in intramuscular connective tissues of Semitendinosus (SD) and Longissimus dorsi (LD).

		6M	9M	12M	18M
Endomysium	SD	1.023 ± 0.063 ^A^	1.098 ± 0.103 ^B^	1.206 ± 0.072 ^C^	2.506 ± 0.025 ^D^
	LD	0.962 ± 0.063 ^A^	1.055 ± 0.074 ^B^	1.196 ± 0.104 ^C^	2.33 ± 0.083 ^D^
Perimysium	SD	0.305 ± 0.030 ^A^	0.314 ± 0.025 ^AB^	0.320 ± 0.021 ^B^	0.704 ± 0.052 ^C^
	LD	0.291 ± 0.018 ^A^	0.305 ± 0.022 ^A^	0.398 ± 0.028 ^B^	0.66 ± 0.048 ^C^

Data are presented as mean ± standard deviation. Different capital letters in the same row indicate significant differences (*p* < 0.05).

## Data Availability

The data presented in this study are available on request from the corresponding author.

## Supplementary Materials

The following supporting information can be downloaded at: https://www.mdpi.com/article/10.3390/ani13030395/s1, Table S1: qRT-PCR primer sequences.
